# Artificial Intelligence in Periodontology: From Automated Diagnosis to Prediction and Clinical Decision Support—A Narrative Review

**DOI:** 10.3390/dj14080531

**Published:** 2026-08-20

**Authors:** Marco M. Herz, Valentin Bartha

**Affiliations:** 1 Department for Conservative Dentistry, Tübingen University, University Hospital of Tübingen, Osianderstr. 2-8, 72076 Tübingen, Germany; 2 Department of Cariology, Endodontology and Periodontology, University of Leipzig, Liebigstraße 12, Haus 1, 04103 Leipzig, Germany; valentin.bartha@medizin.uni-leipzig.de; 3 Department for Conservative Dentistry, Heidelberg University, Im Neuenheimer Feld 400, 69120 Heidelberg, Germany

**Keywords:** artificial intelligence, periodontitis, risk assessment, decision support systems, clinical, dental radiography

## Abstract

**Background/Objectives**: We aimed to evaluate the methodological quality, translational limitations, and clinical applicability of current artificial intelligence (AI) applications in periodontology and to propose a framework for validated prediction and decision support. **Methods**: A structured narrative review based on a targeted, non-systematic literature search was conducted using PubMed and cross-disciplinary sources (January 2015–April 2026). Evidence from primary studies, systematic reviews, and methodological guidance for AI prediction models and clinical decision-support systems was synthesized with a focus on clinical applicability. **Results**: Current periodontal AI research is dominated by retrospective studies focusing on radiographic phenotyping, where deep learning models demonstrate promising diagnostic performance for detecting and quantifying periodontal bone loss. However, substantial limitations persist, including heterogeneous endpoints, inconsistent reporting, limited external validation, and insufficient calibration assessment. Importantly, there is little evidence that AI-based tools improve clinical decision-making or patient-relevant outcomes. Emerging work on prognostic modeling and multimodal data integration highlights the potential for individualized periodontal risk prediction but remains undervalidated, with limited evidence for clinical implementation. **Conclusions**: Although AI-based models show promising diagnostic performance, translational progress in periodontology is currently limited by insufficient validation and the lack of evidence for clinical utility. Future research should prioritize clinically actionable prediction models, robust external validation, and prospective evaluation of AI-supported decision-making within real-world periodontal care pathways.

## 1. Introduction

Periodontal diseases are among the most prevalent chronic inflammatory conditions worldwide and impose substantial burdens on oral function, quality of life, and health systems [1,2]. In clinical practice, diagnosis and monitoring depend on periodontal probing, clinical attachment loss, bleeding on probing, and radiographic bone level assessment—measures that remain central to clinical assessment but often act as retrospective descriptors of destruction rather than as reliable individualized predictors of future trajectories [3,4].

The 2017 World Workshop classification reframed periodontitis as a heterogeneous disease with variable progression and risk, captured through staging and grading [3,4]. This paradigm shift from descriptive diagnosis toward individualized risk assessment provides a particularly suitable conceptual framework for the application of artificial intelligence in periodontology. At the biological level, periodontitis emerges from nonlinear host–microbiome interactions within a dysbiosis framework influenced by systemic and behavioral modifiers [5]. This complexity further limits the ability of static, low-dimensional assessments to capture individual disease trajectories, treatment response, and future risk.

Artificial intelligence (AI) offers computational methods that can learn patterns from large and heterogeneous datasets, complementing (not replacing) clinical reasoning [6,7,8].

Current periodontal AI literature has heavily emphasized automated diagnosis—especially deep learning for radiographic bone loss detection and staging [9,10,11]. While diagnostic automation may reduce variability and improve scalability, the broader clinical opportunity lies in using AI for prediction, risk stratification, monitoring, and decision support along the periodontal care pathway [12,13]. However, evidence for these broader clinical applications remains limited, and most existing models lack prospective validation and integration into real-world clinical workflows. Although recent reviews have comprehensively summarized AI applications in periodontology, they have largely focused on diagnostic performance and evidence synthesis [13,14]. Consequently, important questions regarding clinical translation, implementation, and decision support remain insufficiently addressed.

The present review therefore evaluates the translational limitations of current AI applications in periodontology and examines their potential to evolve from automated phenotyping toward validated prognostic modeling and clinical decision support within longitudinal care pathways. Particular emphasis is placed on distinguishing technical performance from clinical utility and on integrating clinical, radiographic, behavioral, and biological data to support individualized prediction. By adopting this translational perspective, the review proposes a conceptual framework for moving beyond automated diagnosis toward clinically validated prediction and decision-support systems.

## 2. Methods

This narrative review was conducted to critically evaluate the current evidence on artificial intelligence (AI) applications in periodontology, with particular emphasis on their translational relevance for clinical prediction and decision support. The review followed a structured narrative approach, emphasizing transparent literature identification, critical appraisal, and conceptual synthesis.

A targeted, non-systematic literature search was performed using PubMed as the primary database and was complemented by manual searches of reference lists, citation tracking of key publications, and cross-disciplinary methodological literature. The search covered publications from January 2015 to April 2026, reflecting the period during which machine learning and deep learning became increasingly established in periodontal research. Search terms combined periodontal concepts (“periodontitis”, “periodontal bone loss”, “alveolar bone level”, “tooth loss”, “supportive periodontal therapy”) with AI-related concepts (“artificial intelligence”, “machine learning”, “deep learning”, “convolutional neural network”, “prediction model”, “clinical decision support”, “electronic dental record”, and “electronic health record”). Additional publications were identified through backward and forward citation tracking of landmark studies, systematic reviews, consensus reports, and methodological guidance documents.

Because the objective of this review was conceptual synthesis and critical interpretation rather than quantitative evidence aggregation, no formal systematic review methodology or meta-analysis was undertaken. Instead, studies were selected according to their scientific quality, clinical relevance, and contribution to understanding the translational development of AI in periodontology. Preference was given to original investigations, systematic reviews, meta-analyses, international consensus reports, reporting guidelines, and methodological publications addressing clinically meaningful endpoints, external validation, calibration, model transparency, implementation challenges, and clinical decision-support systems. Only publications available in English were considered.

Technical proof-of-concept studies focusing exclusively on algorithm development without clear clinical relevance were included only when they substantially contributed to understanding current methodological capabilities or limitations. Particular attention was paid to studies evaluating automated diagnosis, prognostic prediction, multimodal data integration, personalized risk assessment, and clinical decision support. To facilitate critical appraisal, the discussion was structured according to internationally recognized methodological frameworks for AI in healthcare, including TRIPOD+AI, PROBAST+AI, DECIDE-AI, SPIRIT-AI, and CONSORT-AI. Specifically, TRIPOD+AI provides guidance for transparent reporting of AI-based prediction models, PROBAST+AI supports structured assessment of risk of bias and applicability, and DECIDE-AI focuses on the early-stage clinical evaluation of AI-supported decision-support systems, whereas SPIRIT-AI and CONSORT-AI provide recommendations for the design and reporting of prospective AI intervention studies. These frameworks served as conceptual reference standards for assessing the methodological quality and translational maturity of current periodontal AI research rather than as formal eligibility criteria [15,16,17,18,19].

The purpose of this review was not to provide an exhaustive summary of all published AI applications in periodontology. Rather, it aimed to synthesize the current evidence within a translational framework that emphasizes the progression from automated diagnosis to validated prediction models and clinically applicable decision-support systems integrated into longitudinal periodontal care pathways, while distinguishing technical performance from meaningful clinical utility [6,7,13].

## 3. Current Evidence and Translational Framework

In periodontology, AI can be understood as an enabling layer that transforms heterogeneous inputs into clinically interpretable outputs. Most current applications use supervised learning to perform classification (e.g., bone loss present/absent; stage categories) or regression (e.g., predicted percentage bone loss), while deep learning excels at high-dimensional imaging tasks [6,20]. However, clinical value depends less on the specific algorithmic class than on whether the model is embedded into a clinically meaningful care pathway: diagnosis → risk stratification → treatment planning → monitoring and adaptation [7,13]. This longitudinal and prevention-oriented care pathway makes periodontology particularly well suited for AI-supported prediction, as treatment decisions and maintenance intervals are repeatedly adapted according to changes in individual disease activity and risk.

Before discussing the methodological and translational implications of these developments, it is useful to briefly summarize the principal domains in which AI has been investigated in periodontology. As outlined in Table 1, current research encompasses automated radiographic diagnosis, prognostic prediction from electronic dental records, biomarker-based approaches, and emerging clinical decision-support systems. These application domains provide the evidence base for the subsequent critical appraisal of methodological quality, clinical utility, and implementation readiness.

First, technical performance should not be equated with clinical utility: excellent discrimination in retrospective datasets does not establish that an AI system improves clinical decision-making, patient outcomes, or health-care delivery in routine periodontal practice [21,22]. Second, generalizability should be regarded as a prerequisite for clinical implementation rather than merely a methodological consideration. Differences in imaging devices, acquisition protocols, patient populations, and periodontal charting practices may substantially influence model performance. Without external validation and calibration across diverse clinical settings, even highly accurate models may fail to generalize to routine periodontal care [23,24]. Third, interpretability should serve a clinical purpose: explanations are most valuable when they support clinician oversight, facilitate error detection, and enhance confidence in AI-assisted periodontal decision-making, rather than being viewed as stand-alone guarantees of correctness [25,26].

**Figure 1:**
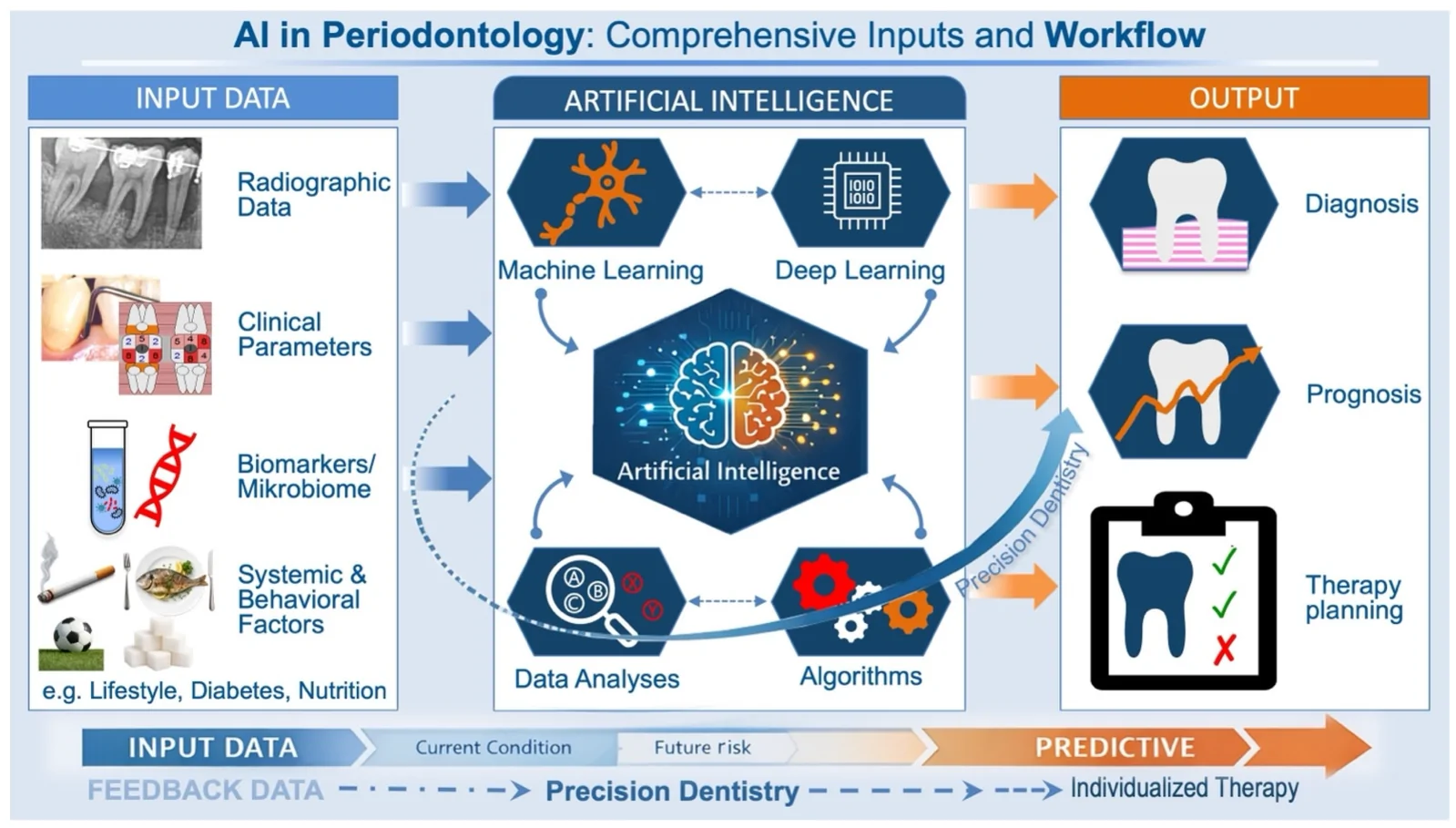
Conceptual framework of artificial intelligence applications across the periodontal care pathway.

Because periodontal AI is transitioning from experimental studies to potential chairside tools, the field benefits from established methodological frameworks that support transparent reporting, bias appraisal, and rigorous clinical evaluation (e.g., TRIPOD+AI, PROBAST+AI, DECIDE-AI, SPIRIT-AI/CONSORT-AI). For prediction modeling, reporting guidance such as TRIPOD+AI and bias/applicability assessment with PROBAST+AI support transparent comparison across models and reduce over-optimistic reporting [15,16,23]. For decision support, DECIDE-AI outlines reporting items for early-stage clinical evaluation, and SPIRIT-AI/CONSORT-AI define how AI interventions should be specified and evaluated in trials [17,18]. As shown in Figure 1, the most clinically relevant view of AI in periodontology is not as a stand-alone diagnostic classifier, but as a care-pathway layer linking phenotyping, risk estimation, and treatment adaptation.

Clinical charting (probing, attachment levels, bleeding), patient-level modifiers (systemic conditions, behaviors), radiology-derived phenotypes (panoramic/periapical bone loss quantification), and biological signals (saliva/GCF biomarkers) feed AI models (ML/DL) to produce standardized diagnostic outputs and individualized risk estimates. The framework emphasizes the transition from one-time diagnostic labeling toward longitudinal risk updating and decision support embedded in supportive periodontal therapy scheduling and treatment adaptation [7,13]. The figure illustrates this conceptual framework, positioning AI as an integrative layer within longitudinal periodontal care rather than a stand-alone diagnostic tool. This concept closely reflects the principles of modern periodontal care, in which diagnosis represents only the starting point of a continuous process including risk assessment, individualized therapy, supportive periodontal treatment (SPT), and long-term monitoring. Consequently, the clinical value of AI in periodontology should ultimately be judged by its ability to support these longitudinal care decisions rather than by diagnostic accuracy alone.

### 3.1. Automated Diagnosis: What AI Can Already Do Reliably—And What It Cannot Yet Prove

Radiographic bone loss detection and quantification is the most established periodontal AI domain. Landmark primary studies demonstrated that deep learning models can detect periodontal bone loss on panoramic radiographs and support tooth-level classification consistent with clinical staging concepts [10,11]. Related work also explored deep learning for diagnosis and prediction of periodontally compromised teeth using CNN-based approaches [9]. Collectively, these studies support a credible claim: in controlled research settings, AI can standardize image-derived periodontal phenotyping and produce reproducible quantitative descriptors that could feed downstream risk models. In periodontology, these standardized imaging phenotypes are particularly valuable because radiographic bone loss represents a core component of staging and longitudinal assessment, providing an objective basis for individualized risk estimation.

However, systematic reviews caution against equating promising diagnostic performance with readiness for routine clinical deployment. A systematic review in JADA concluded that evidence for AI-based periodontal bone loss assessment and classification is limited and of low quality, and that a clear clinical advantage cannot yet be recommended [27]. A more recent Dentomaxillofacial Radiology systematic review using the APPRAISE-AI appraisal tool similarly reported broadly good diagnostic performance across studies but substantial variation in transparency and methodological quality, emphasizing the need for stronger reporting standards and validation [24,28].

Taken together, the available evidence indicates that imaging-based AI is currently the periodontal application closest to clinical translation. Nevertheless, technical maturity should not be equated with clinical readiness, as important evidence gaps remain with respect to external validation, calibration, prospective evaluation, and demonstration of clinical benefit. Current limitations include heterogeneous labeling standards, limited multicenter external validation, insufficient calibration assessment, and the absence of prospective evidence demonstrating clinical benefit. Collectively, these limitations underscore the need to shift the focus from automated diagnosis toward prediction and clinical decision support, where the added value of standardized imaging phenotypes can be evaluated using clinically meaningful outcomes [13,14].

While these findings demonstrate that AI can standardize radiographic periodontal phenotyping, their clinical relevance ultimately depends on whether such outputs translate into improved longitudinal risk assessment and decision-making. The current evidence landscape across diagnostic, predictive, and biomarker-based applications is summarized in Table 1.

### 3.2. Prognostic Modeling and Risk Stratification: The Highest Potential—And the Largest Validation Gap

An important clinical challenge remains in anticipating the future: predicting which patients and sites will experience future progression, recurrence, or tooth loss, and who may benefit from intensified supportive periodontal therapy or adjunctive interventions. AI methods can model nonlinear interactions and integrate broader variable sets than traditional risk scores, including longitudinal patterns and multimodal features [6,7]. The central opportunity in periodontology is to move from describing established damage toward estimating individualized forward risk with explicit uncertainty. Such predictive approaches are especially relevant in periodontology, where long-term disease control depends on individualized supportive periodontal therapy, timely intervention, and prevention of future attachment loss and tooth loss. Unlike many acute conditions, periodontal therapy requires repeated reassessment over many years. This longitudinal disease course provides a unique opportunity for AI to support dynamic risk estimation and individualized adaptation of maintenance strategies.

A representative example is the development of prediction models from large electronic dental record (EDR) datasets. Patel and colleagues used EDR data to develop and test a machine-learning model (XGBoost) for periodontal disease prediction in a cohort exceeding 27,000 patients, leveraging a broad feature set extracted from routine records [12]. Systematic evidence is also emerging: Polizzi and colleagues reviewed AI models for predicting periodontitis, situating the field within risk assessment and personalized planning, while underscoring heterogeneity in study designs and endpoints [14].

This promise comes with a familiar risk: the “accuracy trap.” Prediction model research across medicine has repeatedly shown that many models are at high risk of bias and poorly reported, with limited external validation and unclear usefulness in practice [23]. Periodontal prediction models face similar threats: outcome definitions vary (progression vs. tooth loss vs. recurrence), time horizons are inconsistent, confounding and missingness are common in EHR/EDR data, and calibration (the trustworthiness of predicted probabilities) is often not reported. The field is promising, but the standards must be high: prognostic AI should be regarded as a promising yet under-validated domain, with TRIPOD+AI reporting, PROBAST+AI appraisal, and external validation as minimum thresholds before prediction tools can credibly influence periodontal care pathways [15,16].

## 4. Discussion

### 4.1. Decision Support, Therapy Planning, and Monitoring: Evidence-Informed Optimism

Overall, the available evidence indicates that current periodontal AI research remains of limited clinical relevance unless it progresses beyond diagnostic replication toward demonstrable clinical decision impact. While radiographic phenotyping represents a technically mature application, its clinical relevance depends entirely on integration into longitudinal risk assessment and treatment decision pathways. This highlights a fundamental distinction: accuracy in retrospective datasets does not equate to improved patient care [21,22]. This distinction may be particularly important in periodontology because therapeutic success is not defined solely by accurate diagnosis, but by maintaining periodontal stability over time. Decisions regarding recall intervals, supportive periodontal therapy, retreatment, and adjunctive interventions are repeatedly adapted according to individual risk, making periodontology especially well suited for AI-assisted prediction and decision support.

The translational challenge is therefore not primarily algorithmic, but methodological and clinical. Prediction models must be externally validated, well-calibrated, and evaluated against clinically meaningful endpoints, including disease progression, recurrence, and tooth retention [15,23]. Moreover, AI systems should be assessed as interventions within care pathways, requiring prospective evaluation of usability, safety, and decision impact before broader implementation [17,19]. Therefore, early clinical evaluations should follow established guidance: specify intended use and user roles, document human–AI interaction, characterize error cases and failure modes, evaluate usability and safety in live settings, and only then proceed to pragmatic trials—with SPIRIT-AI/CONSORT-AI supporting protocol and trial reporting, and DECIDE-AI supporting the early-stage clinical evaluation phase [17,18,19]. In periodontology, this approach is particularly relevant because long-term outcomes (progression, tooth retention) require longitudinal study designs; the translation timeline must therefore be explicitly planned rather than assumed.

### 4.2. Bias, Transparency, and Implementation Barriers: Why Translation Is Still Uncommon

**Figure 2:**
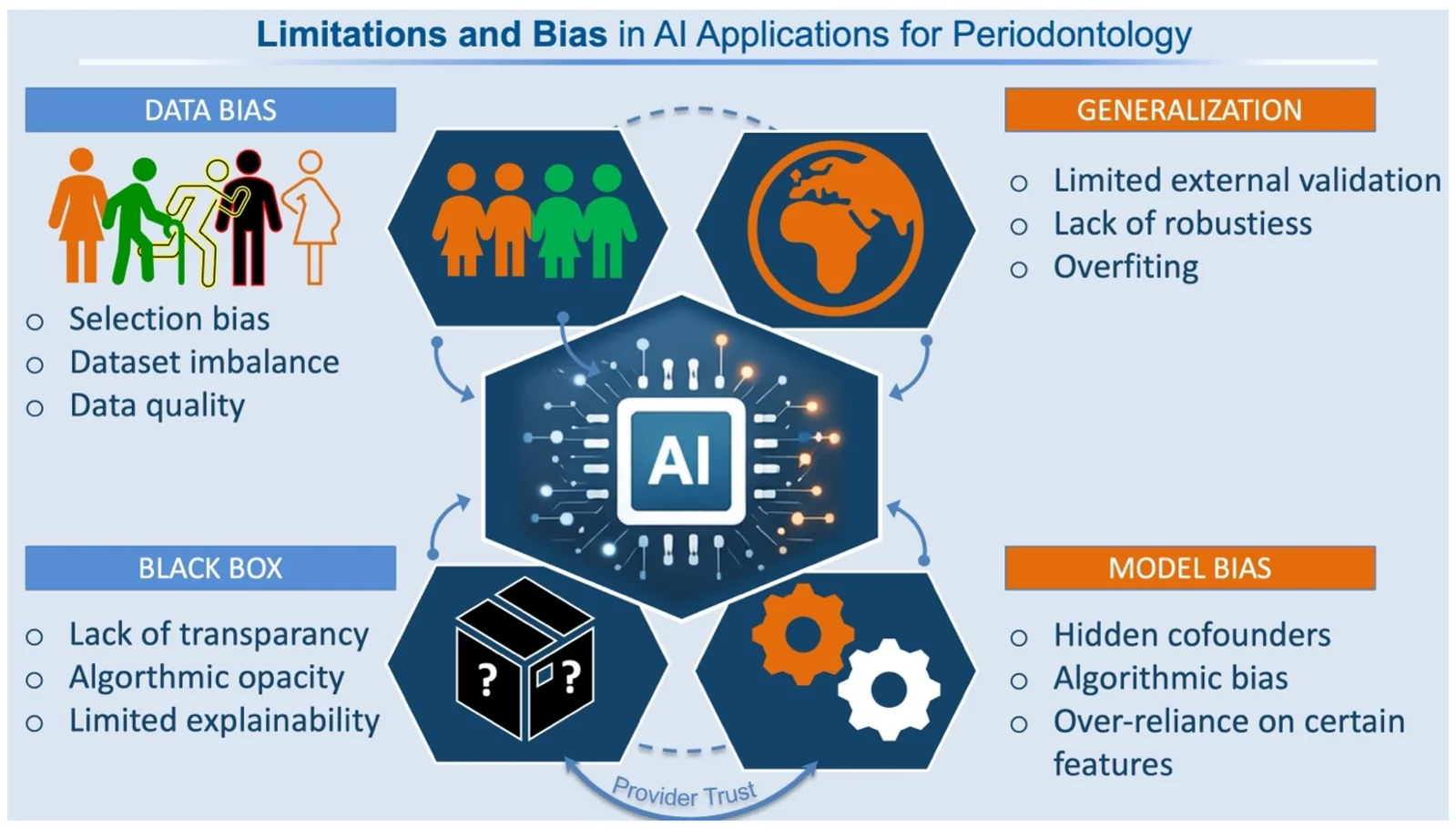
Sources of bias and fragility in periodontal AI models.

The gap between periodontal AI prototypes and routine clinical tools is explained by layered barriers spanning data, modeling, evaluation, and governance (Table 2; Figure 2). Data barriers are foundational: many periodontal AI datasets are retrospective, single-center, and shaped by device-specific imaging pipelines or local care patterns. This can create dataset shift and inequitable performance across populations [24,26]. Across domains, translation is consistently limited by retrospective study designs, heterogeneous endpoints, and insufficient external and prospective validation, which together restrict the clinical credibility and generalizability of current AI models.

Methodological and reporting barriers are equally important. Across medical imaging AI, external validation and head-to-head comparisons against clinicians using the same test set are often absent, and reporting limitations reduce interpretability of performance claims [22]. Periodontal diagnostic AI reviews echo similar shortcomings and emphasize variability in study quality and transparency [24,27]. For prognostic models, bias and poor reporting have been repeatedly documented in other clinical domains, motivating structured reporting and appraisal tools [15,16,23].

Implementation and human factors are often decisive. Even a well-validated model may fail if it does not fit periodontal workflows, increases chairside burden, creates alert fatigue, or leads to automation bias. Interpretability frameworks provide useful conceptual guidance, but real-world safety depends on careful specification of intended use, user training, uncertainty communication, and human oversight [21,25,29]. For periodontal AI, these barriers should be treated not as “later concerns,” but as design constraints that shape model development from the start. These barriers are not isolated technical issues but interacting factors that influence whether periodontal AI can generalize safely across populations and workflows (Figure 2; Table 2).

Although most clinically validated AI applications in periodontology currently rely on two-dimensional radiographs, cone-beam computed tomography (CBCT) represents a promising future imaging modality for AI-assisted assessment of periodontal bone defects. The three-dimensional nature of CBCT may enable more detailed characterization of alveolar bone morphology and complex defect configurations. However, routine periodontal use remains limited by radiation exposure, cost, and current guideline recommendations. Consequently, robust evidence supporting AI-based CBCT applications for routine periodontal diagnosis and risk prediction is still scarce and requires further investigation.

In addition, regulatory and medico-legal considerations will be critical for clinical translation. In the European context, AI-based decision-support systems may fall under medical device regulation, requiring clear definition of intended use, performance, and risk management. Importantly, clinical responsibility remains with the treating clinician, and AI outputs should be interpreted as supportive rather than directive, with appropriate oversight and accountability mechanisms.

Bias can arise at multiple stages: data selection (sampling bias, imbalance), labeling (measurement error, inconsistent staging), modeling (overfitting, shortcut learning), and evaluation (data leakage, absent external validation, poor calibration). The figure highlights that transparency and appraisal tools (e.g., APPRAISE-AI; PROBAST+AI) complement standard performance reporting and help identify translation risks [16,24,28].

### 4.3. Research Agenda and Clinical Translation: Prioritized Steps and Actionable Recommendations

Because periodontal care is inherently longitudinal and preventive, future AI research should prioritize individualized risk assessment and treatment adaptation with the ultimate goal of maintaining periodontal stability and tooth retention, rather than focusing solely on improvements in diagnostic accuracy. A clinically credible roadmap for periodontal AI should prioritize the following research steps:

1. Define standardized, clinically actionable endpoints and horizons for periodontal prediction (e.g., periodontal stability, progression requiring treatment escalation, recurrence during supportive periodontal therapy, and tooth loss risk), and report calibration/decision thresholds explicitly [14,15].
2. Build multicenter, longitudinal datasets linking charting, radiographic phenotypes, systemic modifiers, and lifestyle-related data, including dietary and behavioral factors; pre-specify strategies for handling missingness and label noise [12,24].
3. Require external validation and transportability testing as routine—not exceptional—before any periodontal AI tool is considered for clinical guidance [22,23].
4. Harmonize reporting and critical appraisal: use TRIPOD+AI for prediction models and PROBAST+AI for bias/applicability assessment; use APPRAISE-AI when evaluating clinical decision-support AI study quality [15,16,28].
5. Transition from retrospective performance claims to prospective clinical evaluation and pragmatic trials, following DECIDE-AI for early-stage evaluations and SPIRIT-AI/CONSORT-AI for AI intervention trials [17,18,19].

### 4.4. Practical Recommendations for Clinical Translation

First, adopt a “predict → validate → evaluate” pathway: no chairside recommendations without external validation and calibration, and without transparent reporting that enables replication and auditing [15,16].

Second, design periodontal AI as decision support with explicit human oversight: present quantitative outputs with uncertainty, embed plausible explanations for auditing, and test usability and safety in live settings before scaling [17,25].

Third, measure outcomes that matter: evaluate impact on recurrence/progression, tooth retention, and patient-centered outcomes, and include implementation outcomes (time, acceptability, equity), not only diagnostic accuracy [21,27]. 

**Figure 3:**
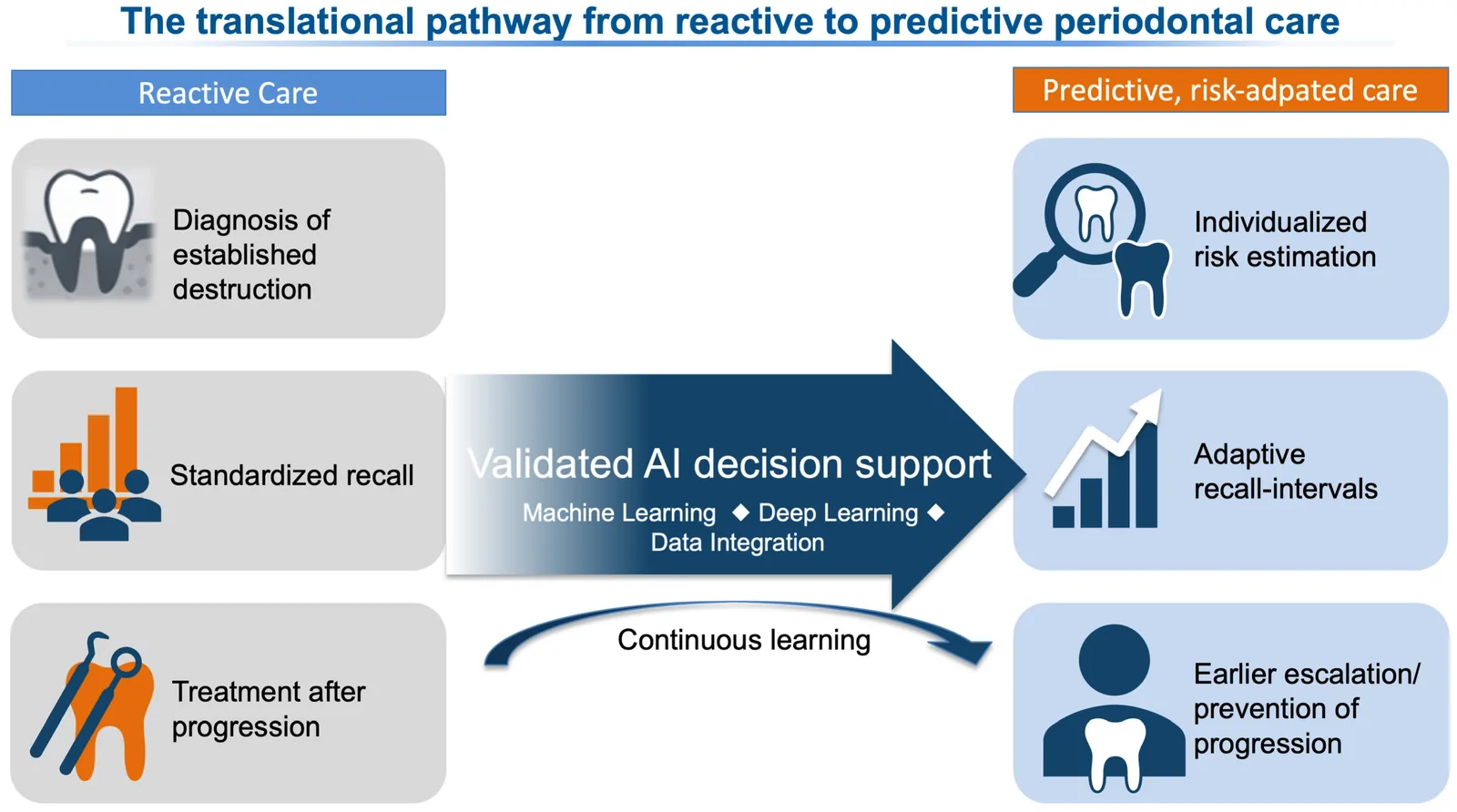
The translational pathway from reactive to predictive periodontal care.

Together, these recommendations define the pathway from reactive periodontal management toward validated, predictive, and risk-adapted periodontal care, as illustrated in Figure 3.

Future periodontal AI should therefore be evaluated not only against conventional diagnostic metrics, but also according to its ability to improve long-term disease stability, tooth retention, patient-centered outcomes, and the efficiency of supportive periodontal therapy.

## 5. Conclusions

Artificial intelligence has demonstrated credible capability in standardizing radiographic periodontal phenotyping; however, current evidence remains insufficient to support routine clinical use. The greatest clinical potential lies in the development of validated predictive and decision-support systems that integrate multimodal data to enable individualized, risk-adapted periodontal care across the entire disease course, from diagnosis to supportive periodontal therapy. Given the chronic and recurrent nature of periodontitis, AI is likely to provide its greatest clinical value not at the time of diagnosis, but throughout long-term periodontal maintenance by supporting individualized, risk-adapted care.

Without rigorous validation, transparent reporting, and prospective demonstration of clinical benefit, improvements in diagnostic performance alone are unlikely to translate into meaningful patient outcomes. The future impact of AI in periodontology will not be determined by increasingly accurate diagnostic algorithms but by the successful development, validation, and implementation of clinically actionable prediction and decision-support systems.

Conventional management is often reactive—detecting and treating established destruction—whereas AI-enabled frameworks could support earlier risk-based stratification, individualized maintenance intensity, and adaptive monitoring based on longitudinal evidence. The figure emphasizes that the shift is not guaranteed by automation alone, but depends on external validation and prospective evidence of clinical benefit and safety [17,21,23].

## Figures and Tables

**Table 1:** Current applications of artificial intelligence in periodontology: domains, endpoints, limitations, and evidence maturity.

Domain	Data Type	Model Type (Typical)	Clinical Endpoint (Typical)	Key Limitations (Typical)	Clinical Readiness
Radiographic detection of periodontal bone loss	Panoramic radiographs	CNN classifiers; patch-based DL; tooth-level classification pipelines	Bone loss present/absent; tooth-level severity	Retrospective datasets; variable annotation standards; limited external validation; device/center shift [1,2]	Development/internally validated
Automated staging/diagnostic mapping from radiographs	Panoramic radiographs	Hybrid DL + conventional image processing/CAD	Tooth-level classification supporting staging concepts	Potential shortcut learning; inconsistent ground truth; limited generalizability evidence [3]	Development/internally validated
Diagnostic quantity synthesis (review-level)	2D dental radiographs (panoramic/periapical)	Systematic review/meta-analysis; quality appraisal with AI-specific tools	Summary sensitivity/specificity/accuracy across studies	Transparency variability; methodological heterogeneity; quality often intermediate [4,5]	Systematic evidence (low–moderate quality)
Periodontal disease prediction from records	Electronic dental records (EDR)	Gradient boosting (e.g., XGBoost); feature-based ML	Patient-level disease category/risk classification	Label noise; missingness; transportability and calibration uncertainties [6]	Development/internally validated
Evidence synthesis for predictive AI	Mixed data types (records ± clinical variables)	Systematic review of prediction studies	Accuracy of AI models in periodontitis prediction	Heterogeneous endpoints and designs; limited external validation evidence [7]	Systematic evidence (low quality)
Personalized diagnostics narrative synthesis	Multimodal (clinical, imaging, molecular)	Narrative review framing of personalized diagnostics	Diagnostic/personalization opportunity	Conceptual breadth; implementation constraints; evidence maturity varies [8]	Conceptual/emerging
Biomarker-based diagnosis/stratification	Saliva/GCF; immunologic or microbiome profiles	Classical ML (RF/SVM/XGBoost); feature selection	Diagnosis/classification from non-invasive biomarkers	Small cohorts; batch effects; limited replication; unclear incremental value over clinical exam [9]	Exploratory
Decision support/therapy planning/monitoring	Longitudinal multimodal data (clinical, radiographic, behavioral, dietary, biological)	Clinical decision-support systems; ML/multimodal predictive models	Risk-adapted recall, escalation decisions, adjunctive therapy selection, and longitudinal progression monitoring	Sparse prospective evidence; unclear usability and workflow integration; limited demonstration of decision impact [7,12,13,17,18,19]	Emerging/conceptual

**Table 2:** Barriers to clinical implementation of periodontal AI (categories and examples).

Barrier Category	Examples in Periodontology	Practical Mitigation Strategies
Data representativeness and dataset shift	Single-center cohorts; device- and protocol-specific radiographs; under-represented subgroups	Multicenter data; external validation; subgroup reporting; drift monitoring [4,10]
Label quality and uncertainty	Variable clinical charting; inconsistent radiographic “ground truth”; EDR coding errors	Adjudication; standardized labeling protocols; uncertainty-aware modeling; sensitivity analyses
Reporting and transparency	Incomplete description of datasets, splits, and evaluation; limited reproducibility	TRIPOD+AI; methodological checklists; structured reporting [11]
Bias and over-optimism in prediction modeling	Overfitting; optimistic internal validation; limited calibration; poor transportability	PROBAST+AI appraisal; external validation; calibration reporting [12,13]
Clinical utility gap	“High accuracy” without evidence of decision impact or outcomes	Prospective “silent mode” testing; clinical utility studies; pragmatic trials [14,15]
Human factors and workflow fit	Chairside time constraints; alert fatigue; automation bias	Human-centered design; uncertainty communication; training; audit trails [16,17]
Ethical and governance concerns	Equity/fairness; accountability for errors; privacy	Fairness audits; governance model; patient communication; oversight [18]
Trial readiness and evaluation standards	AI intervention insufficiently specified; inadequate reporting	SPIRIT-AI protocol guidance; CONSORT-AI reporting guidance [19,20]

## Data Availability

No new data were generated or analyzed in this study. Data sharing is not applicable to this article.

## Abbreviations

The following abbreviations are used in this manuscript:

AI	Artificial Intelligence
APPRAISE-AI	Appraisal of AI Studies—Bewertungsinstrument zur qualitativen Beurteilung von KI-Studien
AUC	Area Under the (ROC) Curve
BMJ	*British Medical Journal*
CNN	Convolutional Neural Network
CONSORT-AI	Consolidated Standards of Reporting Trials—Artificial Intelligence Extension
DECIDE-AI	Reporting guideline for the early-stage clinical evaluation of Decision support systems driven by Artificial Intelligence
DL	Deep Learning
EDR	Electronic Dental Record
EHR	Electronic Health Record
GCF	Gingival Crevicular Fluid
GPT	Generative Pre-trained Transformer
JADA	*Journal of the American Dental Association*
JAMA	*Journal of the American Medical Association*
ML	Machine Learning
MME	Master of Medical Education
MMH	Marco M. Herz (Autorenkürzel)
PROBAST	Prediction model Risk Of Bias ASsessment Tool
PROBAST+AI	PROBAST-Erweiterung für KI-basierte Prädiktionsmodelle
PSD	Polymicrobial Synergy and Dysbiosis (Model)
SPIRIT-AI	Standard Protocol Items: Recommendations for Interventional Trials—Artificial Intelligence Extension
TRIPOD+AI	Transparent Reporting of a multivariable prediction model for Individual Prognosis Or Diagnosis—AI Extension
USA	United States of America
VB	Valentin Bartha (Autorenkürzel)
WA	Washington (Bundesstaat, USA; in der Autorenadresse von Microsoft)
